# Domestication influences morphological and physiological responses to salinity in *Brassica oleracea* seedlings

**DOI:** 10.1093/aobpla/plz046

**Published:** 2019-08-09

**Authors:** M Lema, Md Y Ali, R Retuerto

**Affiliations:** 1 Department of Functional Biology, Faculty of Biology, University of Santiago de Compostela, Santiago de Compostela, Spain; 2 Agrotechnology Discipline, Life Science School, Khulna University, Khulna, Bangladesh

**Keywords:** *Brassica* crops, domesticated-wild, Na^+^ exclusion, salt stress, succulence

## Abstract

*Brassica oleracea* cultivars include important vegetable and forage crops grown worldwide, whereas the wild counterpart occurs naturally on European sea cliffs. Domestication and selection processes have led to phenotypic and genetic divergence between domesticated plants and their wild ancestors that inhabit coastal areas and are exposed to saline conditions. Salinity is one of the most limiting factors for crop production. However, little is known about how salinity affects plants in relation to domestication of *B. oleracea*. The objective of this study was to determine the influence of domestication status (wild, landrace or cultivar) on the response of different *B. oleracea* crops to salinity, as measured by seed germination, plant growth, water content and mineral concentration parameters at the seedling stage. For this purpose, two independent pot experiments were conducted with six accessions of *B. oleracea*, including cabbage (group *capitata*) and kale (group *acephala*), in a growth chamber under controlled environmental conditions. In both taxonomic groups, differences in domestication status and salt stress significantly affected all major process such as germination, changes in dry matter, water relations and mineral uptake. In the *acephala* experiment, the domestication × salinity interaction significantly affected water content parameters and shoot Na^+^ allocation. At early stages of development, wild plants are more succulent than cultivated plants and have a higher capacity to maintain lower Na^+^ concentrations in their shoots in response to increasing levels of salinity. Different responses of domesticated and cultivated accessions in relation to these traits indicated a high level of natural variation in wild *B. oleracea*. Exclusion of Na^+^ from shoots and increasing succulence may enhance salt tolerance in *B. oleracea* exposed to extreme salinity in the long term. The wild germplasm can potentially be used to improve the salt tolerance of crops by the identification of useful genes and incorporation of these into salinity-sensitive cultivars.

## Teaser

*Brassica oleracea* cultivars include important vegetable and forage crops grown worldwide, whereas the wild counterpart occurs naturally on European sea cliffs. Understanding how non-domesticated plants that survive in saline environments manage sodium toxicity will be useful for determining the mechanism of stress tolerance in *B. oleracea* and could help to identify useful traits to improve salt tolerance in *Brassica* crops. This study reveals that wild *B. oleracea* has evolved various adaptation mechanisms, such as exclusion of sodium from shoots and increasing succulence, to cope with high salinity levels in the long term.

## Introduction

Exposure of plants to salt stress has become a threat to human civilization as it reduces the yield of most crops, which limits the goal of higher food production for the world’s ever-growing population. Also of concern is the fact that many areas are increasingly affected by salinization, mainly in intensively cropped and densely populated areas of the world. According to the FAO (2017), ~800 million hectares of land are currently affected by salinization, and as a result 0.25–0.50 million hectares of agricultural land are lost every year. The main constraint to production is that very few crops are salt-tolerant, and cultivars of particular crop species vary greatly in relation to this trait (Greenway and Munns 1980; Ashraf and Sarwar 2002).

Crop plants are generally glycophytic (i.e. sensitive to high salinity), although cabbage (*Brassica oleracea* var. *capitata*) is considered moderately sensitive to salinity (Bernstein *et al.* 1974). Kale (*B. oleracea* var. *acephala*) reaches acceptable growth rates and can tolerate saline irrigation during early stages of growth, and is therefore a potential candidate for use in drainage water reuse systems (Shannon *et al.* 2000). Wild *B. oleracea* grows in saline environments, inhabiting sea cliffs, where it cannot evade the effects of salt. The species is therefore expected to have developed at least some degree of salt resistance, using different mechanisms to avoid (salt regulation by exclusion, elimination, dilution and/or compartmentalization) or tolerate the effects produced by the increased concentrations of ions. Significant morphological and physiological differences between domesticated and wild accessions have been found in maize (Chinchilla-Ramirez *et al.* 2017) and also in *B. oleracea*, among other crop species, under artificially imposed stress (Matesanz and Milla 2018).

Genetic diversity in relation to salt tolerance and other traits originally present in wild *B. oleracea* has been reduced in modern cultivars due to human-mediated selection, which has caused several genetic bottlenecks. However, it can be assumed that some classes of alleles, in many cases with minor effects, may have been missed during domestication and/or crop improvement and remain unexploited in modern cultivars (Prada 2009). It is therefore important to explore how salt tolerance has changed between ancestors (wild forms) and current domesticate genotypes (landraces and modern cultivars). The main difference between landraces (local varieties) and cultivars is that landraces are grown in agricultural ecosystems, whereas cultivars arise as the result of formal breeding programmes. Most modern cultivars descend from a relatively small number of founder landraces (Prada 2009). Consequently, the genes controlling agronomic characters in current cultivars retain low levels of diversity relative to the entire gene pool (landraces and wild forms) from which they arise. For this reason, domestication status may be a determining factor in salt tolerance.

Cultivated *B. oleracea* includes important vegetable and forage crops grown throughout the world, representing, together with cereals, the basis of world supplies (Cartea *et al.* 2011). Farmers have selected different forms of domesticated *B. oleracea* to produce crops with distinct uses and characteristics: broccoli, Brussels sprouts, cabbage, cauliflower and kale, among others. The diversification of *B. oleracea*, caused by selection processes, has led to differentiation of several botanical varieties or groups, such as *italica*, *gemmifera*, *capitata*, *botrytis* and *acephala*, corresponding, respectively, to the above-mentioned crops (Branca and Cartea 2011). Different *B. oleracea* crops have been demonstrated to display high phenotypic and genetic diversity (Cartea *et al.* 2003; Padilla *et al.* 2007; Branca 2008; Vilar *et al.* 2008), probably in relation to regional preferences, resulting in numerous choices of edible forms. Despite the relatively high variability in *B. oleracea* crops, the genetic variation could be further increased to maintain and extend the cultivation of *Brassica* species in suboptimal land affected by salt stress. According to Arzani and Ashraf (2016), the *in situ* genetic resources that have enabled the plants to adapt to survive and grow in saline habitats are crucial to increase the genetic variability in relation to salinity tolerance in crops. Investigation of whether such variation is found in wild *Brassica* and exploration of the underlying mechanisms of salinity tolerance are decisive for advancement in breeding programmes and increased production in saline environments.

Domesticated (commercial or elite cultivars, germplasm and landraces) and wild forms could be crossed to broaden the genetic base and improve the salt tolerance of *B. oleracea* crops. Breeders should be aware of the high cost of recovering the genetic background of the receptor elite cultivar when wild species are used to enhance salt tolerance (Cuartero *et al.* 2006). Nevertheless, recovering the background of the elite cultivar from crosses between kale and wild *B. oleracea* may be feasible, taking in account that kale may represent an ‘evolutionary bridge’ for the other taxonomical groups included in *B. oleracea* (Branca and Cartea 2011).

For most crop species, several evaluation and selection programmes have led to the establishment of germplasm collections. Conventional breeding programmes and other more innovative approaches, such as the use of *in vitro* selection, interspecific hybridization, marker-assisted selection, transformation, mutation and polyploidy, have been used to enhance salt tolerance in different crops (Flowers *et al.* 1997; Flowers 2004; Cuartero *et al.* 2006). However, salt-tolerant cultivars remain scarce, probably due to the quantitative nature of this trait, in which numerous genes have small effects. Several authors have demonstrated the existence of variations in salt tolerance among species belonging to the genus *Brassica* (Ashraf *et al.* 1999, 2001; Ashraf and McNeilly 2004; Agarwal *et al.* 2008; Kumar *et al.* 2009) and also within some *Brassica* species (Maggio *et al.* 2005; Ali *et al.* 2006) and crops (Sanoubar *et al.* 2016). For crop improvement, evaluation of salt sensitivity in all growing phases has been recommended to identify and prioritize efforts on the most salt-sensitive developmental stage (Cuartero *et al.* 2006). The early stage of growth has been recognized as the most vulnerable life stage in plants, as weak seedlings do not reach the reproductive stage under stressful conditions (Chinchilla-Ramirez *et al.* 2017). The initial growth stage will be crucial for stress tolerance, as selective pressures will act on the most relevant seedling traits to overcome the restrictive conditions. Plants that are resistant/tolerant to abiotic stress at initial growth stages will probably show enhanced resistance at later developmental stages.

Research findings in diverse fields suggest that salt tolerance has changed during domestication and selection processes; however, no studies comparing *Brassica* genotypes with contrasting domestication status have been carried out. We aimed to determine whether traits leading to salt tolerance in wild *B. oleracea* that survives on sea cliffs have been lost during domestication and/or improvement processes. Although the effects of soil salinity on the performance of *Brassica* species is well known and widely documented (see above-mentioned references), studies involving accessions belonging to different botanical varieties or groups are scarce. The objective of this study was to determine the influence of domestication status (wild, landrace or cultivar) on the response of *B. oleracea*, including the taxonomic varieties *acephala* (kale) and *capitata* (cabbage), to increasing levels of salinity, as measured by germination, plant growth and allocation traits, and water content and mineral concentration parameters.

## Materials and Methods

### Plant material and growth conditions

For this research, two independent experiments were carried out with the *capitata* (cabbage) and *acephala* (kale) groups of *B. oleracea*. Experiment 1 (‘*capitata* experiment’) was conducted between October 2013 and January 2014 and included cabbage accessions with different domestication status: the commercial and extensively used cultivar ‘Corazon de Buey Grande’ (*capitata* cultivar, Rocalba S.A.), the landrace MBG-BRS535 (*capitata* landrace—a local variety collected from Galegos, Lugo, Spain at 42.9385°N, −7.5685°W) and the wild accession EXT-BRS0151 (wild-*capitata*—collected from sea cliffs in Foz, Lugo, Spain at 43.6279°N, −7.3340°W; Fagundez *et al.* 2015). Experiment 2 (‘*acephala* experiment’) was conducted between September and December 2014 and included kale accessions with different domestication status: the commercial and extensively used cultivar ‘Gigante Caballar’ (*acephala* cultivar, Rocalba S.A.), the landrace MBG-BRS0510 (*acephala* landrace—a local variety collected from Baleira, Lugo, Spain at 43.0285°N, −7.1756°W) and the wild accession EXT-BRS0217 (wild-*acephala*—collected from sea cliffs in Cudillero, Asturias, Spain at 43.5664°N, −6.1478°W). The six accessions were provided by the *Brassica* Genebank held in the Misión Biológica de Galicia (MBG-CSIC), Spain.

In both experiments, plants were maintained under controlled environmental conditions in a plant growth chamber (temperature 24 ± 2 °C, 50–60 % relative humidity, photoperiod of 16 h light and 8 h dark and 500–550 μmol photons m^−2^ s^−1^ of photosynthetically active radiation) belonging to the Department of Functional Biology, University of Santiago de Compostela, Spain.

### Salt treatments

The experiments included four treatments with different concentrations of NaCl in the irrigation water: 0, 50, 100 and 150 mM NaCl. The electrical conductivity (EC) values for 0 (control), 50, 100 and 150 mM NaCl solutions were, respectively, 1.45, 5.15, 9.48 and 13.28 dS m^−1^.

Ten-day-old seedlings grown under non-saline conditions were transferred in 1-L plastic pots with perforated bottom filled with organic substrate (‘Sondermischung’, Gramoflor GmbH & Co. KG). One month after the seedlings were transplanted, sodium chloride was added to the irrigation water. The four treatments were arranged in a completely randomized design. Plants belonging to both groups (*acephala* and *capitata*) were exposed to salt treatments for 3 months. Three times a week, the pots were placed in a tray filled with water with the respective amount of salt, and the soil was allowed to reach 100 % water holding capacity by being soaked for 4 h in the saline solution. The pots were then returned to the culture shelves in the growth chamber.

### Germination

Three replications of 20 seeds per accession were germinated in Petri dishes on filter paper soaked with sodium chloride solution (50, 100 and 150 mM NaCl) or non-saline water (control—0 mM NaCl). Seed germination was evaluated daily, and those seeds in which the radicle had emerged through the seed coat were considered to have germinated. The germination percentage was recorded after 10 days. The mean germination time (MGT = −∑Dn/∑n, where ‘n’ is number of seeds emerged on day, and ‘D’ is the number of days from sowing to the emergence) was calculated according to Abbas *et al.* (2013). In the *capitata* experiment, after 10 days none of the seeds of the wild accession were able to germinate at 150 mM.

### Plant growth and water content parameters

At the end of the experiments, all plants (*n* = 240, 10 plants by 4 treatments and 6 accessions) were de-potted along with substrate. The roots were then carefully washed with flowing tap water to remove the soil. The plants were separated into roots and shoots (leaves and stems), and the fresh weight was measured with a digital balance (Mettler Toledo mod. ML203/01, 220 ± 0.001 g, Columbus, OH, USA). The plant parts were then oven-dried separately at 40 °C for at least 7 days to constant weight, and the root and shoot dry weights were measured. The values of root mass ratio (RMR = root mass/plant mass), shoot mass ratio (SMR = shoot mass/plant mass) and total water content [TWC = (leaf fresh mass − leaf dry mass)/leaf fresh mass] were calculated. Salinity response models were constructed and salt-tolerance index (ST-index) was calculated for each accession, according to Steppuhn *et al.* (2005a, b). For this purpose, relative yield (Y_r_) was calculated by dividing the absolute yield (Y, total plant dry mass) by dry mass under control conditions (0 mM NaCl), when salinity has no influence on mass production (Y_m_). To test the response to salinity, different models were chosen for each accession on the basis of the best-fit line and highest *r*^2^ (Steppuhn *et al.* 2005b). The ST-index, proposed by Steppuhn *et al.* (2005a) as an indicator of the inherent tolerance of agricultural crops to salt stress, was calculated as ST-index = EC_e50_ (1 + b), where EC_e50_ is the electrical conductivity of the root zone that reduces the relative yield to 50 % of the absolute yield (EC_e_ at Y_r_ = 0.5) and ‘b’ is a regression constant. EC_e_ was derived from the electrical conductivity of the irrigation water solutions (EC_i_) by the relationship EC_e_ = 0.5 EC_i_.

Before the plants were harvested, two leaf discs of 1.8 cm diameter were punched from the uppermost fully expanded leaf on each plant and were immediately weighed to determine the fresh mass. The discs were then placed in a 5-cm diameter Petri dish containing distilled water, for 24 h at 4 °C in darkness, to obtain the saturated mass. Once the fully turgid leaf discs were weighed, they were oven-dried and again weighed to determine the dry mass. The succulence degree [SD = (turgid mass − dry mass)/leaf area, in g dm^−2^], relative water content [RWC = (fresh mass − dry mass)/(turgid mass − dry mass) * 100], specific leaf area (SLA = leaf area/dry mass, in dm^2^ g^−1^) and saturated water content [SWC = (turgid mass − dry mass)/dry mass] were determined according to Ogburn and Edwards (2012).

### Mineral concentration

The roots and shoots of all harvested plants were dried in oven at 40 °C for 7 days, before being ground to a fine powder in a ball mill (Retsch® model MM400, Verder Holding B.V., Vleuten, GG, The Netherlands), digested with nitric acid and extracted by an ultrasound method. Samples (10 mg) were placed in 10 mL of HNO_3_ (0.2 N; 0.5 % Tritin X-100) for ultrasonic-assisted pseudo-digestion with an ultrasonic processor (SONICS Vibra-Cell™ model VCX 130, Sonics & Materials, Inc., Newtown, CT, USA), operated during 60 s at 80 % power. The concentrations of Na^+^ and K^+^ were determined in liquid phase by flame atomic absorption spectrometry (Perkin Elmer mod. 2100, Norwalk, CT, USA), and the corresponding concentration in the plant tissue was calculated (expressed as percentage dry mass). For analytical quality control, duplicate samples were analysed once every nine samples, and analytical blanks were used to control for possible contamination. Certified reference material (‘poplar leaves: GBW07604’) was also analysed once every nine samples.

Data on germination and plant growth and allocation traits were obtained in both experiments (six accessions, including cabbage and kale). Plant water content and mineral concentration were also determined in kale accessions. The *acephala* group (Experiment 2) was selected for the above-mentioned analyses because the growth habit and organ morphology are similar to those of wild types. Furthermore, this group represents an ‘evolutionary bridge’ between wild types and the other taxonomic groups in *B. oleracea* (Branca and Cartea 2011). The greatest differences relative to other taxonomical groups were expected to be observed in water content and mineral concentration.

### Statistical analysis

All data used in this study are included as Supporting Information (see File S1 to S4). Data from *acephala* and *capitata* experiments were analysed separately. Two-way analysis of variance (ANOVA) was used to evaluate the effects of salinity (0, 50, 100 and 150 mM NaCl) and domestication status (cultivar, landrace and wild), and their interaction, all of which were included in the model as fixed-effect factors. For each trait, Fisher’s protected least significant difference (LSD) was used to compare mean values, at a significance level of 0.05 (Steel *et al.* 1997). Regression analysis was used to model different response functions relating relative yield of *B. oleracea* accessions to root-zone salinity. Phenotypic correlation coefficients between traits associated with mineral content, plant growth and water content parameters were calculated. All analyses were performed using SAS® 9.4 software and JMP® 12.1.0 (SAS Institute Inc., Cary, NC, USA).

## Results

The ANOVA revealed significant effects of domestication status (*P* ≤ 0.046) on germination, growth and allocation traits and TWC in both experiments (Table 1). Salinity significantly affected germination traits in both experiments, as well as root dry mass (*P* = 0.044) and TWC (*P* < 0.0001) in *acephala* and SMR and RMR in *capitata* (Table 1). Similar responses to salinity were observed in domesticated and wild accessions as the domestication status × salinity interaction was not significant for any of the above-mentioned variables, except germination traits (Table 1).

**Table 1.** Result of the two-way ANOVA assessing the effect of salinity on germination, growth and allocation traits and TWC of six accessions of *Brassica oleracea* with different domestication status (cultivar, landrace and wild) and representing *acephala* (kale) and *capitata* (cabbage) taxonomic groups. ns, not significant. *Significant at *P* < 0.05; **P < 0.01; ***P < 0.001. The numbers of degrees of freedom (df) for germination and growth/allocation traits were, respectively, 36 and 112.

	Domestication status (df = 2)	Salinity (df = 3)	Domestication status × salinity (df = 6)
*Brassica oleracea acephala*			
Seed germination (%)	485.0***	25.7***	6.22***
MGT (days)	74.1***	36.7***	18.2***
Total plant dry mass (g)	8.15***	1.88 ns	0.49 ns
Shoot dry mass (g)	8.66***	1.70 ns	0.61 ns
Root dry mass (g)	8.13***	2.79*	0.40 ns
SMR	18.9***	1.45 ns	1.78 ns
RMR	18.9***	1.46 ns	1.78 ns
TWC	7.32***	21.97***	0.38 ns
*Brassica oleracea capitata*			
Seed germination (%)	269.6***	15.2***	5.19**
MGT (days)	78.0***	10.4**	2.59 ns
Total plant dry mass (g)	6.27**	1.10 ns	0.55 ns
Shoot dry mass (g)	7.26**	1.11 ns	0.65 ns
Root dry mass (g)	3.09*	1.68 ns	0.35 ns
SMR	3.83*	3.01*	2.08 ns
RMR	3.83*	3.01*	2.08 ns
TWC	3.29*	0.95 ns	1.07 ns

In the *acephala* experiment, the ANOVA revealed that domestication status significantly affected all water content parameters and mineral concentrations, except for shoot K^+^ concentrations and shoot K^+^/Na^+^ ratio (Table 2). In addition, salinity significantly affected all traits except shoot K^+^ concentrations. Domestication groups differed widely in their response to salinity as regards RWC (*P* < 0.0001), SLA (*P* = 0.0001) and SD (*P* = 0.0098) (Table 2).

**Table 2.** Result of the two-way ANOVA assessing the effect of salinity on water content parameters and mineral concentration of three accessions of *Brassica oleracea* with different domestication status (cultivar, landrace and wild) and representing the *acephala* (kale) taxonomic group. ns, not significant. *Significant at *P* < 0.05; **P < 0.01; ***P < 0.001. The number of degrees of freedom (df) for water content parameters and mineral concentration traits were 239 and 59, respectively.

	Domestication status (df = 2)	Salinity (df = 3)	Domestication status × salinity (df = 6)
Water content parameters			
RWC	13.4***	86.3***	7.84***
SWC	10.8***	4.75**	1.82ns
SLA (dm^2^ g^−1^)	24.7***	6.91***	4.77***
SD (g dm^−2^)	39.6***	41.1***	2.89**
Mineral concentration			
Root K^+^	3.89*	13.9***	0.80ns
Root Na^+^	8.62***	13.1***	1.46ns
Root K^+^/Na^+^ ratio	3.72*	9.11***	0.31ns
Shoot K^+^	0.29ns	1.54ns	2.03ns
Shoot Na^+^	6.30**	52.5***	2.36*
Shoot K^+^/Na^+^ ratio	2.18ns	15.4***	1.3ns

### Germination

**Figure 1.**
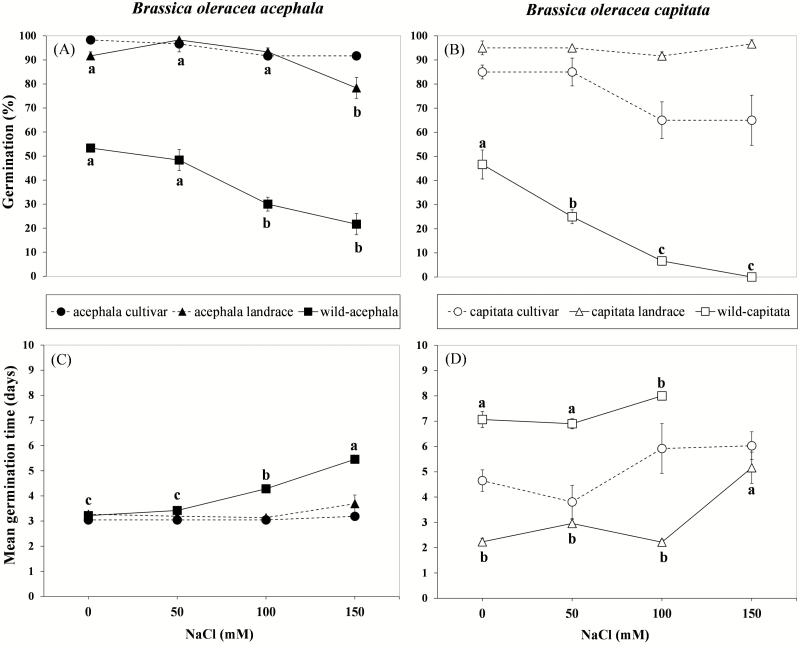
Effect of increasing salt concentrations (0, 50, 100 and 150 mM NaCl) on percentage of germination and MGT in *Brassica oleracea acephala* (A, C) and *B. oleracea capitata* (B, D) experiments including accessions with different domestication status (cultivar, landrace and wild). Data are means of three measurements per domestication status and salinity level (error bars = SE). Significant salinity × domestication status interactions are indicated by solid lines; non-significant interactions are indicated by dashed lines (*P* > 5 %, LSD test). Means indicated by equal letters are not significantly different (*P* > 5 %, LSD test).

The effect of salinity on seed germination percentage depended on domestication status in the *acephala* (*P* < 0.001) and *capitata* (*P* < 0.001) experiments (Table 1). While in the different landraces and cultivars, the germination percentage was not affected by salinity treatment (except in the *acephala* landrace), in the wild accessions seed germination percentage decreased significantly as the salinity increased (Fig. 1A and B). Mean germination time varied significantly in relation to domestication status (Table 1), with the highest values corresponding to wild accessions, indicating a positive effect (i.e. reduction in MGT) of domestication on this character (Fig. 1C and D). Salinity increased the MGT (Table 1), although in the *acephala* experiment this effect depended on the domestication status, and only the wild accession was significantly affected by salinity (Fig. 1C).

### Plant growth and allocation traits

**Figure 2.**
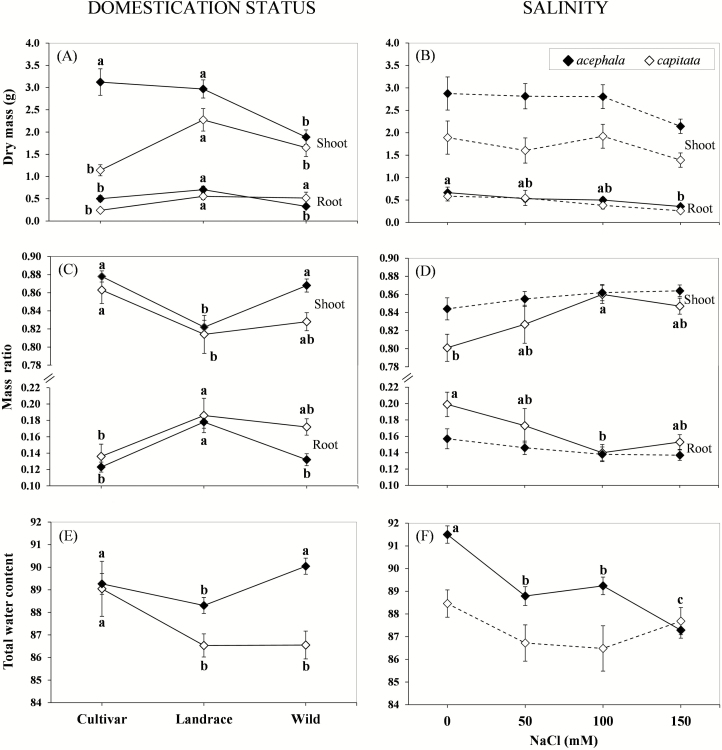
Effect of domestication status (A, C, E) and increasing salinity (B, D, F) on growth, allocation and TWC of domesticated (landraces and cultivars) and non-domesticated (wild) *Brassica oleracea* accessions belonging to the *acephala* and *capitata* taxonomic groups. Data are means of 40 and 30 measurements per domestication status and salinity, respectively (error bars = SE). Significant effect of salinity on growth, allocation and TWC are indicated by solid lines; non-significant effects are indicated by dashed lines (*P* > 5 %, LSD test). Means indicated by equal letters are not significantly different (*P* > 5 %, LSD test).

Domestication significantly affected total, shoot and root dry mass (*P* always ≤ 0.049; Table 1; Fig. 2A). Although salt stress reduced total, shoot and root dry mass in both experiments, significant differences were only observed for root dry mass (*P* = 0.044; Table 1) in the *acephala* group (Fig. 2B). The reduction in root dry mass was greater for exposure to 150 mM NaCl, but the values were statistically similar to those of plants irrigated with 50 and 100 mM NaCl. For all dry mass measurements, all domestication groups responded similarly to salt stress, as revealed by the absence of a significant domestication status × salinity interaction.

In both experiments, accessions differed significantly in their RMR (*P* always < 0.025) and SMR (*P* always < 0.025) (Table 1). According to LSD comparisons, landraces dedicated a higher proportion of total biomass to roots than the wild accessions and cultivars (Fig. 2C), although in *capitata* the values were similar for the wild accession and landrace (17 and 18 %, respectively). Shoot mass ratio increased gradually with increasing saline stress in both experiments. However, the salinity increased significantly the above-ground dry matter (*P* = 0.034), and the RMR consequently decreased (*P* = 0.034), only in *capitata* (Table 1; Fig. 2D).

The TWC was lowest in the landraces, which differed significantly from cultivars in both experiments and also from the wild accessions in *acephala* (*P* < 0.041; Table 1; Fig. 2E). In addition, in plants irrigated with saline water, the TWC was significantly lower than in control plants in the *acephala* experiment (*P* < 0.0001; Fig. 2F). A similar trend was observed in *capitata*, but the differences in response to the different salinities were not significant (*P* = 0.421; Fig. 2F).

**Figure 3.**
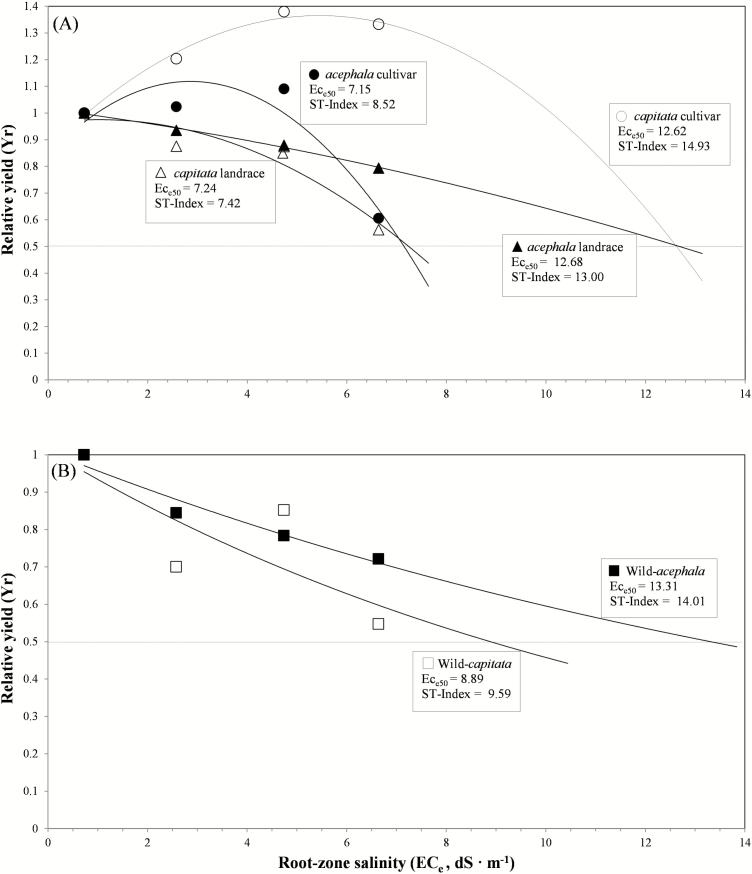
Yield response to increasing root-zone salinity of (A) domesticated (cultivars and landraces) and (B) non-domesticated (wild) *Brassica oleracea* accessions. Landraces and cultivars are categorized into *acephala* (kale) and *capitata* (cabbage) taxonomic groups. Wild accessions are named according to the experiment where they were included. Each point represents the mean total dry mass of 10 plants of each accession.

Additional information about intra-taxonomic group variation can be obtained by analysing the relative yield of all *B. oleracea* accessions as a function of salinity in domesticated (Fig. 3A) and non-domesticated (wild) accessions (Fig. 3B). The quadratic model [Y_r_ = a + (b * EC_e_) + (c * EC_e_^2^)] and the exponential model [Y_r_ = a * exp(b * EC_e_)] provided the best-fit lines and highest *R*^2^ values for domesticated accessions (cultivars and landraces) and wild accessions, respectively (Table 3). The values of EC in the root zone that reduced the yield to 50 % of the maximum (EC_e50_) and the ST-index are also shown in Fig. 3. In general, the relative yield decreased with increasing salt concentration, except for those cultivars in which an initial stimulatory effect was induced by salt (Fig. 3A). The highest ST-index values corresponded to the *capitata* cultivar (14.93), the wild-*acephala* (14.01) and the *acephala* landrace (13.00). By contrast, the lowest values of this index were obtained for the *capitata* landrace, *acephala* cultivar and wild-*capitata* (7.42, 8.52 and 9.59, respectively).

**Table 3.** Coefficient of determination (*R*^2^) and root-mean-square error (RMSE) and parameters (a = constant that reflects the shape of the curve, and b = defines the intensity of the model) from regression analyses relating relative yield and root-zone salinity in six accessions of *Brassica oleracea* with different domestication status (cultivar, landrace and wild) and representing the *acephala* and *capitata* taxonomic groups.

Accession	*R* ^2^	RMSE	a	b
*Brassica oleracea acephala*				
*acephala* cultivar (kale)	0.8689	0.1377	0.8445	0.1917
*acephala* landrace (kale)	0.9922	0.0134	1.0150	0.0243
wild-*acephala*	0.9509	0.0334	1.0087	0.0520
*Brassica oleracea capitata*				
*capitata* cultivar (cabbage)	0.9857	0.0349	0.8676	0.1828
*capitata* landrace (cabbage)	0.8951	0.1047	0.9623	0.0252
wild-*capitata*	0.6135	0.1495	1.0110	0.0790

### Water content parameters

**Figure 4.**
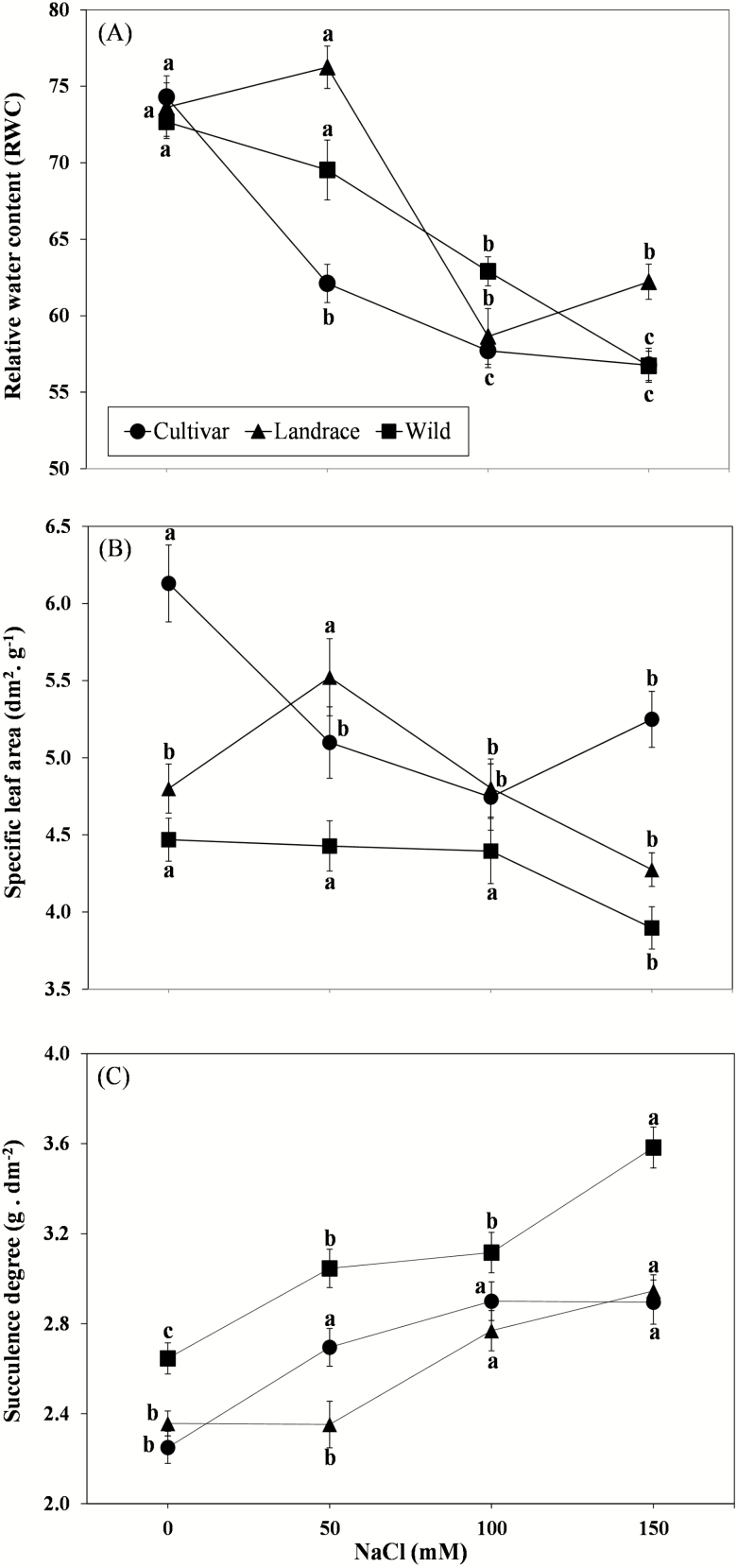
Changes in (A) RWC, (B) SLA and (C) SD with increasing saline content of the irrigation water (0, 50, 100 and 150 mM NaCl) in leaves of three accessions of *Brassica oleracea acephala* with different domestication status (cultivar, landrace and wild). Data are means of 10 measurements per domestication status and salinity (error bars = SE). Means indicated by equal letters are not significantly different (*P* > 5 %, LSD test).

Highly significant differences (*P* < 0.001; Table 2) between domestication status, salt treatments and their interaction were detected for RWC, SLA and SD in the *acephala* experiment. The interaction was not significant (*P* = 0.096; Table 2) for SWC. Increasing concentrations of NaCl in the irrigation water caused a significant reduction (*P* < 0.001) in RWC in all three domestication groups (Table 2). In the highest salt treatment, the RWC value was highest in the *acephala* landrace, differing from *acephala* cultivar and wild plants (Table 4; Fig. 4A). The highest SWC was recorded in the *acephala* cultivar (13.94), relative to the wild plants (13.09) and the *acephala* landrace (12.37; LSD = 0.66). Saturated water content tended to increase with salinity (from 12.33 for control conditions to 13.74 for 150 mM NaCl treatment; LSD = 0.77). Salt stress significantly decreased the SLA of all accessions, although the difference between accessions was greatest for the control (0 mM) and at maximum salt concentration (150 mM), with the *acephala* cultivar showing the lowest values across all salt treatments (Fig. 4B). The SD values were higher in wild plants than in domesticated accessions under all experimental conditions (Fig. 4C). However, although the SD of wild plants increased gradually and significantly with increasing salinity, no significant differences were observed between SD for 100 and 150 mM NaCl in the domesticated accessions.

**Table 4.** Mean values of water content parameters and K^+^ and Na^+^ concentrations (in shoots and roots) of three accessions of *Brassica oleracea* with different domestication status (cultivar, landrace and wild) and representing the *acephala* (kale) taxonomic group grown under different salinities (0, 50, 100 and 150 mM NaCl). Mean values indicated by different letters are significantly different (*P* < 0.05) according to LSD test. Mean values shown in italics are not significantly different.

	RWC	SWC	SLA (dm^2^ g^−1^)	SD (g dm^−2^)	Root			Shoot		
					K^+^	Na^+^	K^+^/Na^+^ ratio	K^+^	Na^+^	K^+^/Na^+^ ratio
					(% dry mass)			(% dry mass)		
Domestication status										
Cultivar	62.7c	13.94a	5.31a	2.68b	1.59b	1.00a	2.02b	*3.38*	3.50a	*1.35*
Landrace	67.7a	13.09b	4.85b	2.60b	1.78ab	0.82b	2.75ab	*3.08*	3.80a	*1.15*
Wild	65.4b	12.37c	4.30c	3.10a	2.10a	0.68b	3.90a	*3.25*	2.79b	*1.95*
LSD	1.9	0.66	0.28	0.12	0.38	0.15	1.60	–	0.58	*–*
Salinity										
0 mM	73.5a	12.33b	5.13a	2.42d	2.44a	0.52c	5.83a	*3.41*	0.96c	3.89a
50 mM	69.3b	13.09ab	5.02a	2.70c	2.13a	0.72b	3.22b	*3.09*	2.93b	1.06b
100 mM	59.7c	13.38a	4.65b	2.93b	1.59b	1.02a	1.98bc	*3.66*	4.31a	0.85b
150 mM	58.6c	13.74a	4.47b	3.14a	1.15c	1.01a	1.24c	*2.79*	4.95a	0.62b
LSD	2.2	0.77	0.33	0.13	0.43	0.18	1.86	*–*	0.67	1.05

### Mineral concentration

**Figure 5.**
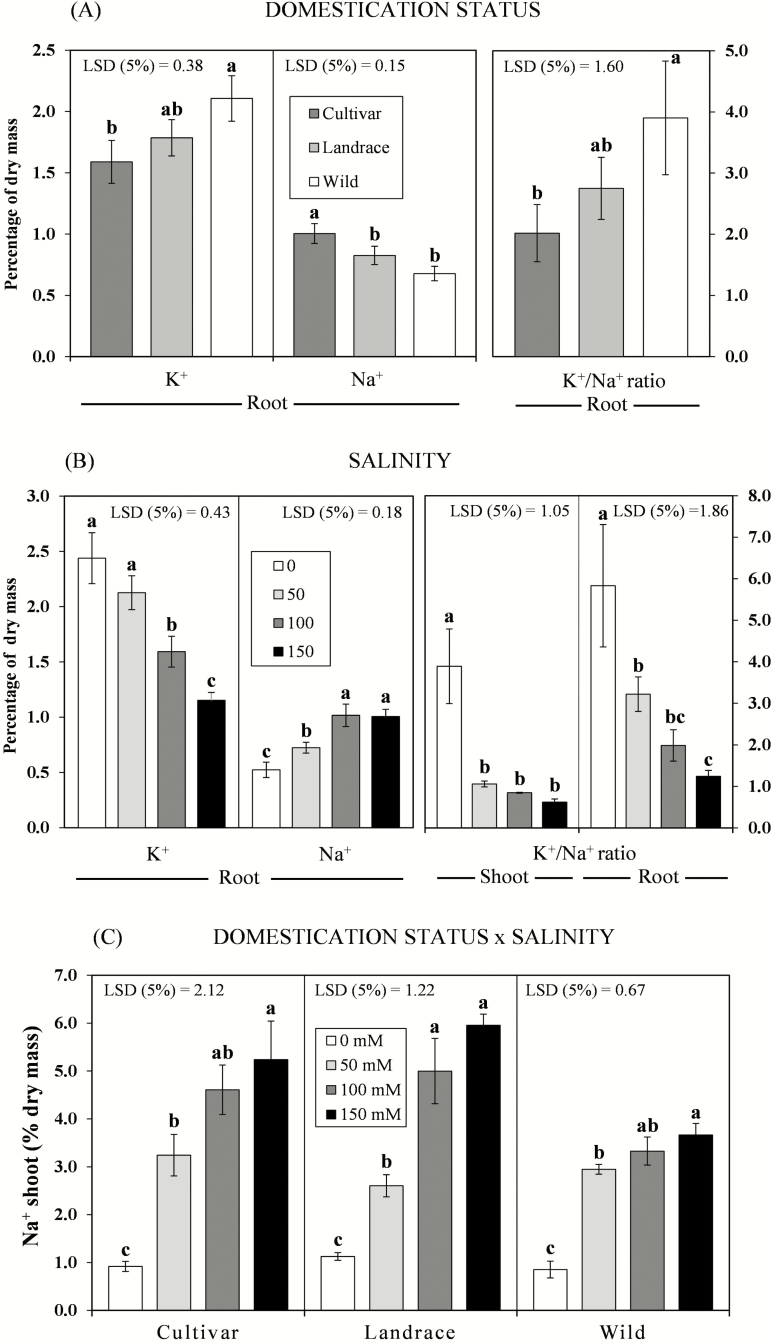
Effect of (A) domestication status (cultivar, landrace and wild) (*n* = 20), (B) increasing salinity (0, 50, 100 and 150 mM NaCl) (*n* = 15) and (C) increasing salt concentrations per domestication status on K^+^ and Na^+^ mean concentrations and K^+^/Na^+^ ratio measured on shoots and roots of plants of three *Brassica oleracea acephala* accessions (*n* = 5) representing domesticated (cultivar and landrace) and non-domesticated (wild) gene pools (error bars = SE). Means indicated by equal letters are not significantly different (*P* > 5 %, LSD test).

In the *acephala* experiment, wild plants contained significantly less Na^+^ in shoots (*P* = 0.0038) and roots (*P* = 0.0007) than the domesticated accessions (Table 2; Fig. 5A). In addition, the root K^+^ content was significantly higher in the wild accession than in the *acephala* cultivar, while the difference relative to the *acephala* landrace was not significant (*P* = 0.027; Fig. 5A). Under saline and non-saline conditions, the mean concentrations of Na^+^ and K^+^ were higher in shoots than in roots in all accessions (Table 4). Differences between accessions were also observed in the root K^+^/Na^+^ ratio: the values were highest in the wild accession but not significantly different from those in the *acephala* landrace (Fig. 5A).

The concentrations of K^+^ decreased and those of Na^+^ increased in roots as the salinity increased, independently of domestication status (Fig. 5B). In addition, the salt treatment significantly decreased the K^+^/Na^+^ ratio in the shoots and roots of all accessions (Fig. 5B).

A significant domestication status × salt treatment interaction (*P* = 0.045; Table 2) was observed for shoot Na^+^, indicating that the wild and cultivated accessions responded differently to increasing concentrations of NaCl in the irrigation water (Table 2; Fig. 5C). Although the behaviour of the accessions was similar at 0 and 50 mM, the wild plants contained significantly less Na^+^ in shoots than both domesticated accessions at higher NaCl concentrations (100 and 150 mM NaCl). Notably, the shoot Na^+^ was slightly higher in the wild accession at above 50 mM NaCl, whereas in the *acephala* landrace and *acephala* cultivar, the shoot Na^+^ increased considerably with increasing salt concentrations (Fig. 5C).

Correlations between traits, based on domestication status across salt treatments, were calculated. Germination percentage was significantly and positively correlated with shoot (*r* = 0.624; *P* = 0.030) dry mass. In addition, succulence was significantly associated with SLA (*r* = −0.795; *P* = 0.002), RWC (*r* = −0.736; *P* = 0.007) and total plant (*r* = −0.624; *P* = 0.030) and shoot (*r* = −0.654; *P* = 0.021) dry mass. Regarding the mineral concentrations, the RWC was positively correlated with root K^+^ (*r* = 0.859; *P* = 0.0003) and shoot K^+^/Na^+^ ratio (*r* = 0.627; *P* = 0.029) and negatively correlated with Na^+^ in shoots (*r* = −0.8168; *P* = 0.0012) and roots (*r* = −0.796; *P* = 0.002). The shoot K^+^/Na^+^ ratio was more strongly influenced by Na^+^ (*r* = −0.796; *P* = 0.0019) than by K^+^ (*r* = 0.285; *P* = 0.3686), but the root K^+^/Na^+^ ratio was influenced to a similar degree by both cations (*r* = 0.919 and *P* < 0.0001 for K^+^; *r* = −0.868 and *P* = 0.0003 for Na^+^).

## Discussion

In the present study, the growth and allocation traits in seedlings of domesticated and non-domesticated (wild) accessions exhibited similar responses to varying saline conditions. However, differences in the relative yield between cultivars and landraces within domestication groups were marked, although expected, as accessions from two different taxonomical groups were compared. In addition, the effects of domestication (transition from wild plants to landraces) and modern breeding (transition from landraces to cultivars) were variable and depended on the trait considered. Domestication has evidently mediated the seed germination and leaf succulence and shoot Na^+^ of seedlings, while modern breeding, along with domestication, may also have influenced the RWC and SLA.

Although we could not reveal how domestication has led to the loss of salt tolerance of an elite cultivar because the phylogenetic relationships among the wild types, landraces and elite cultivars used in this study are unknown, we provide strong evidences that wild types have potential as sources of salt tolerance.

### Germination

We found that increasing salinity significantly affected the time to emergence as well as the final germination percentage. However, the extent of the response varied between domesticated and wild accessions in relation to percentage (in both groups) and time to germination (only in the *acephala* group). The slight reduction in germination for landraces and cultivars contrasted with the abrupt decrease in final germination in wild accessions. Seed mortality at salinity below 150 mM NaCl was not confirmed; however, the pronounced decrease and/or suppression of seed germination at higher salinity levels in wild accessions probably corresponds to an adaptive mechanism described in species surviving in littoral habitats and which is decisive in the ability of such species to colonize saline environments (Ishikawa and Kachi 2000). In a comparison of wild species from contrasting coastal habitats, Mariko *et al.* (1992) found that even under favourable conditions sea cliff species germinate slowly. This probably guarantees germination at the ‘safe site’ and the subsequent establishment of seedlings in these environments. Salt-adapted Brassicaceae species also show high sensitivity to NaCl during germination (Orsini *et al.* 2010). Delayed and decreased germination in wild accessions is not associated with salt tolerance at advanced growing stages, as reported for other crops (Foolad *et al.* 1999; Flowers 2004; Cuartero *et al.* 2006).

In the present study, the salt tolerance during seed germination shown by the domesticated accessions, in terms of higher germination percentage and lower time to germination, may be related to human-mediated selection processes (Fuller 2007). While domestication and selection processes lead to high germination percentage and rapid germination in cultivated genotypes, wild genotypes have evolved adaptations to saline environments, such as increased succulence of leaves. During domestication, wild *B. oleracea* plants were removed from hostile saline habitats, where non-saline water supplies were limited and occasional, to benign farming environments with less restrictive conditions regarding the availability of fresh water. Under the latter conditions, plants were artificially selected for early emergence and high rates of germination over several generations, leading to changes in seed characteristics that resulted in seeds showing loss of dormancy (Fuller and Allaby 2009) (i.e. with a more permeable and thinner seed coat that enables rapid germination even under saline conditions, at least at concentrations reaching 150 mM NaCl). This trait may be undesirable in hostile conditions (i.e. sea cliffs inhabited by the wild type) because it increases the vulnerability of plants to further adverse conditions, but is intentionally selected in plants growing in agricultural environments (i.e. landraces and cultivars grown as crops) (Fuller and Allaby 2009).

### Plant growth and allocation traits

The study findings did not indicate any clear relationship between salinity tolerance and domestication status of the accessions. Contrary to our expectations, the value of the ST-index was lower in the wild-*capitata* accession than in the wild-*acephala* and similar to those of the domesticated gene pool. This finding may be explained by hybridization between cultivated and wild accessions, which is possible as there are some domestic gardens close to sea cliffs in this area. Branca and Cartea (2011) reported evidence of crosses between wild populations of several *Brassica* species growing near villages and *B. oleracea* landraces in Sicily. In the present study, the wild and cultivated accessions belong to the same species, and cross-pollination may be frequent. The wild-*capitata* could survive in its saline natural habitat due to remaining pre-domestication traits such as dormancy (i.e. strong inhibition of germination, especially under adverse conditions). In addition, higher levels of salt tolerance are expected to occur in wild accessions at advanced stages of development.

Salt particularly influences growth processes, so that growth rates and biomass production are reliable criteria for assessing the degree of salt stress in a plant and its salt-tolerance capacity (Flowers and Flowers 2005). In addition, changes in biomass allocation may be determinant in salt tolerance (Byrt and Munns 2008), and shoot growth is generally more salt-sensitive than root growth (Munns 2002; Benincasa *et al.* 2013). It can be assumed that a reduction in leaf area relative to root growth will decrease water uptake, thus conserving soil moisture and preventing osmotic stress due to the salt concentration (Munns and Tester 2008). The present findings show that increased salinity led to a decrease in total dry mass, with a large reduction in root mass. Although significant differences were only observed in one group in relation to root dry mass (*acephala*) and mass ratio (*capitata*), the trends were similar in both groups. The decrease in dry mass between non-saline and the maximum concentration of NaCl (150 mM) in the irrigation water was 56 and 47 % in roots compared to 27 and 25 % in shoots in *capitata* and *acephala*, respectively. Thus, irrespective of domestication status, salinity seems to alter the allocation pattern of biomass, reducing root mass and, consequently, affecting SMR and RMR (Fig. 2C and D). By contrast, Jamil *et al.* (2005) found that salt stress inhibited shoot growth more than root growth in *B. napus* (canola) and *B. oleracea* (cabbage and cauliflower). In field-grown cabbage, Maggio *et al.* (2005) did not find significant differences in shoot/root ratios under several salinity treatments due to similar inhibition in shoot and root growth. Consequently, these authors stated that the shoot/root ratio did not occur as a morphological adaptation to cope with saline conditions in salt-stressed plants.

Munns and Tester (2008) indicated that initial inhibition of cell expansion and lateral bud development would be lower in genotypes exhibiting tolerance to osmotic stress. A reduced response to osmotic stress would produce an increase in leaf area relative to that in plants more susceptible to salinity. This may be an advantage in annual plants growing in environments with no water restrictions (i.e. cultivars and landraces growing in agricultural systems, where higher productivity is recurrent and intentionally selected), but undesirable in perennial plants in which survival prevails over growth (i.e. wild types living on sea cliffs). Accordingly, we found that two domesticated accessions (i.e. the *capitata* cultivar and *acephala* landrace) outperformed the wild accessions. This may indicate a reduced response to osmotic stress in domesticated plants (annual or biannual) in contrast to wild (perennial) accessions, in which productivity is not important. In addition, a higher level of salt tolerance is expected in wild accessions at advanced stages of development (after continued exposure to saline conditions) or when NaCl concentrations are more limiting at initial stages of growth. Matesanz and Milla (2018) also found that under favourable conditions, several cultivated crop plants show enhanced growth and biomass production relative to their wild counterparts, but that this domestication advantage disappears under stressful conditions. This may be of interest in crop breeding when broader adaptation (inclusive for stressful and unpredictable environments) is desirable.

### Water content parameters

Saline conditions affect crop growth patterns and productivity and also modify plant–water relationships, limiting water availability to plants in a similar way to drought stress conditions (Munns and Tester 2008). Water content per unit dry mass (SWC) increases under salt stress. In addition, higher SWC values in the *acephala* cultivar may be related to the lower dry mass of less dense cells and tissues, resulting in a thinner-leaved accession with higher SLA, as leaf traits may be subject to directional selection in order to improve sensory attributes of leaves (i.e. firmness, fibrosity, etc.).

Specific leaf area has been reported to be a good indicator of leaf tissue density and resource use strategies (Barrera Zambrano *et al.* 2014), with light interception for plants with lower SLA being more expensive (Poorter *et al.* 2009). In the present study, the gradual reduction in SLA from wild plants to cultivars may be related to domestication and to the selection processes conducted in modern breeding programmes. Such programmes have aimed to improve productivity and quality and have focused on producing larger amounts of less dense leaves (higher SLA; Fig. 4B), and the genotypes would therefore be more efficient in resource utilization and/or distribution. Specific leaf area was inversely related to succulence (*r* = −0.795; *P* = 0.002), and increased salinity thus induced more succulent and thicker leaves (lower SLA). Although increased succulence (lower SLA) may be considered a costly strategy, in terms of energy consumption, it provides an advantage and proves determinant when ion toxicity is critical and survival becomes more important than growth, mainly in perennial species such as wild *B. oleracea*.

Succulence is related to the capacity of several plant tissues to store water and maintain physiological functions under stressful conditions (Ogburn and Edwards 2012). Succulence enlarges cells (Munns and Tester 2008; Han *et al.* 2013) and increases the number of cell layers (Ottow *et al.* 2005), with subsequent dilution of salt, but no modification of leaf area. Succulence may therefore reduce the effect of ionic and osmotic stress produced by elevated salinity, ameliorating the reproductive ability in saline environments (Agarie *et al.* 2007). *Brassica* crops enhanced salt tolerance via the development of succulent leaves (Ashraf and McNeilly 1990; Maggio *et al.* 2005) and pods (Mogensen *et al.* 1997). Shannon and Grieve (1999) found that under salt stress, cabbage heads are generally more compact and the leaves are fleshier than under non-saline condition.

In the present study, we found that leaf succulence increased with increasing salinity levels, indicating greater water retention in all *B. oleracea acephala* accessions to minimize the toxic effect of salt. Under more saline conditions, wild *B. oleracea* developed much more succulent leaves than in the control treatment (26 % increase, to >3.58 g dm^−2^) and in cultivated accessions (to a maximum SD of 2.90 g dm^−2^ in the *acephala* cultivar and 2.95 g dm^−2^ in the *acephala* landrace). In contrast with domesticated accessions, the wild accession appears to maintain the ability to increase succulence even at the higher salinity level (150 mM NaCl), while the maximum capacity of cultivated plants to retain water is possibly reached at these salt concentrations. This suggests that succulence may indicate lack of domestication as possession of this anatomical trait may have permitted wild *Brassica* to survive in saline habitats, by enhancing their capacity to retain water and dilute the salt content. This anatomical adaptation is also often present in halophytic plants (Munns and Tester 2008; Wang *et al.* 2012).

### Mineral concentration

Sodium (Na^+^) and chloride (Cl^−^) are the main ions responsible for both osmotic and ion-specific damage, which significantly reduces crop growth and yield (Munns and Tester 2008). Osmotic effects occur immediately after salt stress and cause severe alterations to plant water status (Munns and Tester 2008). Ionic stress appears later and causes premature senescence of older leaves and toxicity symptoms (chlorosis, necrosis) in mature leaves (Tester and Davenport 2003; Munns *et al.* 2006), as high Na^+^ concentrations disrupt protein synthesis and interfere with enzyme activity (Blaha *et al.* 2000). Differences in ion partitioning and the maintenance of higher K^+^ to Na^+^ ratios appear to be important mechanisms contributing to salt tolerance and are important selection criteria for identifying salt-tolerant genotypes (Wei *et al.* 2003; Flowers 2004). In the present study, we found significant genotypic differences between cultivar and wild type for the K^+^/Na^+^ ratio in roots, but not in shoots (Table 2), in which the K^+^/Na^+^ values were much lower (mainly due to the higher Na^+^ content, with the shoot K^+^/Na^+^ ratio being irrelevant) similarly to what has been reported for other crops (Wang *et al.* 2012).

Greenway and Munns (1980) established that for optimal efficiency, the K^+^/Na^+^ ratio in glycophytes should be higher than 1.0. We found that this threshold was not reached in shoots of the *acephala* cultivar in any salt treatment or in the *acephala* landrace and wild type from 100 mM NaCl. In roots, the K^+^/Na^+^ value was lower than 1.0 in the *acephala* cultivar from a concentration of 100 mM NaCl, whereas this limit was not lower in the *acephala* landrace and wild accession at any level of salinity (data not shown), demonstrating higher salt tolerance in the wild accession and *acephala* landrace than in the *acephala* cultivar. This suggests that the ability to tolerate salinity may be lost in successive selection processes accomplished by modern breeding programmes aiming to produce new cultivars.

Accumulation (‘tissue tolerance’) and exclusion (by reduction of salt uptake) of toxic ions are both mechanisms responsible for salt tolerance in plants (Munns *et al.* 2015). ‘Tissue tolerance’ is defined by Munns *et al.* (2016) as the capacity of tissues to function while maintaining high internal Na^+^ and Cl^−^ concentrations, mainly through compartmentalization of these toxic ions in vacuoles (‘cellular tolerance’) and using organic and inorganic solutes to achieve osmotic regulation. This strategy is generally manifested in halophytic species and in the most salt-tolerant non-halophytes. Nevertheless, ion exclusion is the most common mechanism used by glycophytes to adapt to saline conditions, as in the short term it is less costly, in terms of energy consumption, than compartmentalization with long-term advantages (Munns and Gilliham 2015). Salt-tolerant genotypes have lower shoot Na^+^ concentrations and higher shoot K^+^ concentrations, and thus higher shoot K^+^/Na^+^ ratios, than the sensitive genotypes in crops such as wheat (Oyiga *et al.* 2016) and barley (Wei *et al.* 2003), demonstrating that the exclusion of Na^+^ on leaves is involved in salt tolerance in these crops. This mechanism is also recognized in *Brassica* species (Ashraf and McNeilly 2004; Kumar *et al.* 2009). In a comparison of the tolerance of three amphidiploid *Brassica* species (*B. juncea*, *B. napus* and *B. carinata*) and their putative diploid relatives (*B. rapa*, *B. nigra* and *B. oleracea*), Ashraf *et al.* (2001) demonstrated that amphidiploid species discriminate for K^+^ and against Na^+^ in ion uptake into the roots and the later translocation to shoots. The amphidiploid species were thus considered more salt-tolerant than the diploids. Ulfat *et al.* (2007) found that in canola (*B. napus*), the most salt-tolerant genotypes have a higher capacity to exclude Na^+^ and Cl^−^ in leaves. By contrast, Ashraf and Ali (2008) did not find any differences in K^+^/Na^+^ ratios in shoots or roots among four canola cultivars with contrasting salt tolerance, and Batool *et al.* (2013) did not find any genotypic differences in mineral concentrations between two cauliflower cultivars under salt stress. In the present study, in all accessions the Na^+^ concentrations increased and K^+^ concentrations decreased in shoots and roots as salinity increased. However, shoot and root Na^+^ concentrations were significantly lower and the root K^+^ and K^+^/Na^+^ ratio were higher in the wild accession than in the cultivated plants, despite the high variability in the latter trait among wild plants. Unlike in domesticated plants, in the wild accession the concentrations of Na^+^ in shoots remained stable independently of variations in salinity, indicating that control of Na^+^ exclusion and transport within the plant may represent an important mechanism for managing salt stress in wild *B. oleracea*.

In cultivated wheat and in wild Triticeae species, salt tolerance is generally correlated with the capacity to maintain low Na^+^ and high K^+^ concentrations in leaves (Colmer *et al.* 2006), although Munns and Tester (2008) reported some exceptions in which salt tolerance and Na^+^ exclusion are not correlated. We did not find a significant correlation between Na^+^ accumulation (in shoot and roots) and salt tolerance (based on dry mass accumulation) in seedlings. Nevertheless, shoot and root mineral concentrations appear to be associated with the ST-index, based on yield reduction under salt stress, which reflects the level of tolerance of the accessions to increasing root-zone salinity. Indeed, strict regulation of Na^+^ transport from the external nutrient solution to the shoot is involved in enhanced salt tolerance in non-domesticated species, in terms of lower levels of biomass reduction and survival, under extreme and/or long-term salinity exposure. Cuartero *et al.* (2002) also reported variations in this trait (among others), indicating that breeding programmes should consider incorporation of the trait as a useful way of increasing salt tolerance in tomato plants. The next steps will be to facilitate the use of wild genetic resources in agriculture and to advance the development of tools for the identification and introgression of useful genes.

## Conclusion

Understanding how non-domesticated plants that survive in saline environments manage sodium toxicity will be useful for determining the mechanism of stress tolerance in *B. oleracea* and could help to identify useful traits to improve salt tolerance in *Brassica* crops. At the initial stages of development, cultivated plants outperformed wild accessions in growth parameters, probably due to their reduced response to osmotic stress. However, wild *B. oleracea* has evolved various adaptation mechanisms to cope with high salinity levels in the long term. Under a prolonged period of salt stress, the leaves of this species developed increased succulence and maintained lower shoot Na^+^ concentration. Differences in regulation of Na^+^ transport from external root nutrient solution to shoots (ion exclusion) and succulence may therefore be important for enhancing salt tolerance under continuous, extreme salinity. Such differences may also be important selection criteria for identifying and developing salt-tolerant genotypes.

## Supporting Information

The data used in this study are available as Supporting Information.

File S1. Germination data.csv

File S2. Plant growth and allocation traits data.csv

File S3. Water content parameters data.csv

File S4. Mineral concentration data.csv

plz046_suppl_Supplementary_Table

plz046_suppl_Supplementary_Figure

## Sources of Funding

M.L. recognizes an Isidro Parga Pondal-I2C Program fellowship from the Xunta de Galicia. Md.Y.A. was supported by the EXPERTS_II (Erasmus Mundus) Interchange Program.

## Contributions by the authors

M.L. and R.R. conceived and design the research experiment. Md.Y.A. and M.L. conducted the experiment, acquiring all data for further analysis. M.L., R.R. and Md.Y.A. analyzed the data and prepared the manuscript. All the authors have read and approved the final manuscript.

## Conflict of Interest

None declared.
