# Supplementary material for: Domestication influences morphological and physiological responses to salinity in Brassica oleracea seedlings
Source: AoB Plants. 2019 Aug 9;11(5):plz046. doi: 10.1093/aobpla/plz046 (PMC6757351; doi:10.1093/aobpla/plz046)
Supplement: plz046_suppl_Supplementary_Figure [file plz046_suppl_supplementary_figure.pdf]

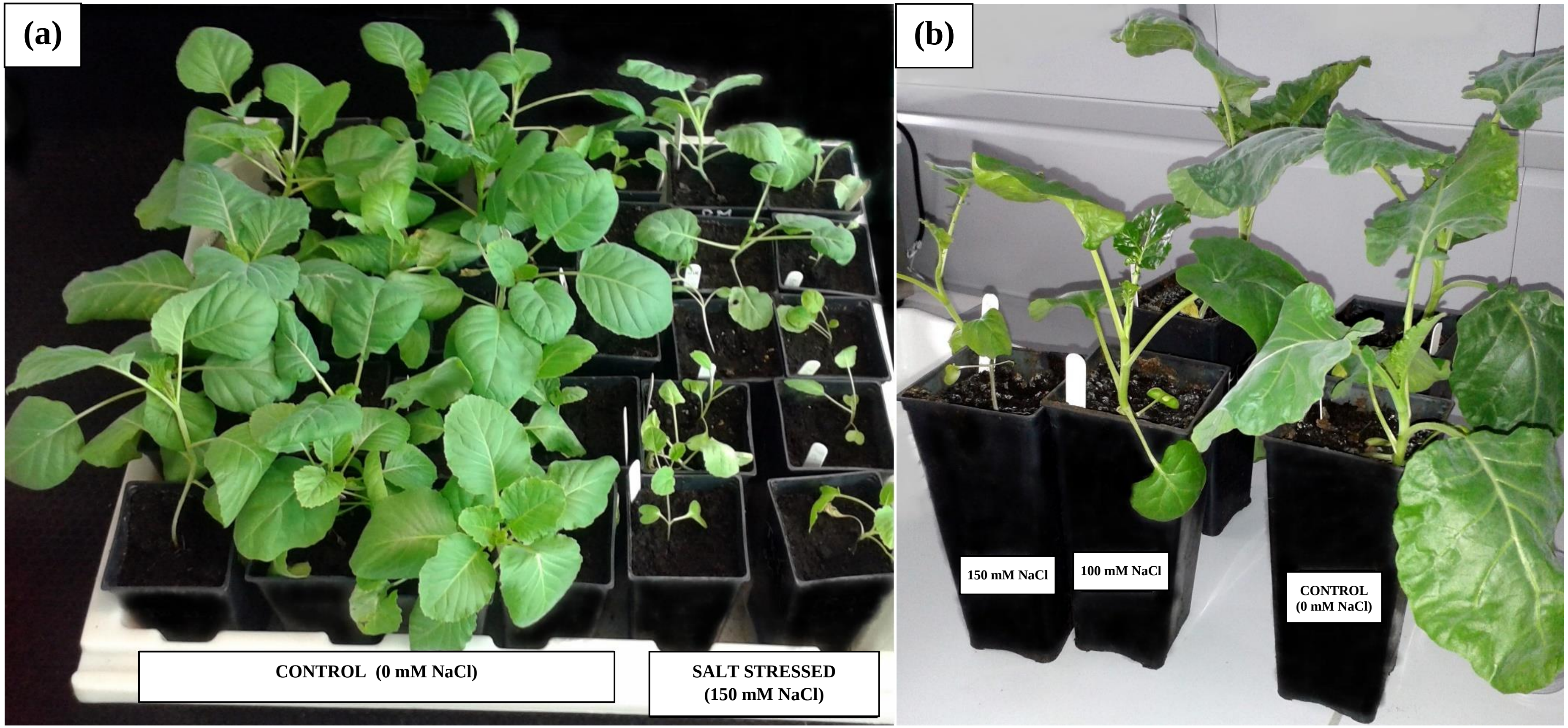

Supplementary material.

Image 1. Contrasting performance of 8-week-old seedlings of a (a) salt-susceptible (capitata landrace) and (b) salt-tolerant (Wild-acephala) *Brassica oleracea* accessions growing under increasing salt concentrations (0, 50, 100 and 150 mM NaCl). Saline irrigation was imposed approx. 4 weeks after sowing by placing the pots (1L) during a 2-hours period (three times per week) in a tray filled with water with the respective amount of sodium chloride.
